# Lower Serum Fibroblast Growth Factor 21 Levels are Associated with Normal Lumbar Spine Bone Mineral Density in Hemodialysis Patients

**DOI:** 10.3390/ijerph17061938

**Published:** 2020-03-16

**Authors:** Yin-Ting Wu, Bang-Gee Hsu, Chih-Hsien Wang, Yu-Li Lin, Yu-Hsien Lai, Chiu-Huang Kuo

**Affiliations:** 1 School of Medicine, Tzu-Chi University, Hualien 970, Taiwan; 103311149@gms.tcu.edu.tw (Y.-T.W.); gee.lily@msa.hinet.net (B.-G.H.); wangch33@gmail.com (C.-H.W.); nomo8931126@gmail.com (Y.-L.L.); 2 Division of Nephrology, Buddhist Tzu-Chi General Hospital, Hualien 970, Taiwan; hsienhsien@gmail.com; 3 School of Post-baccalaureate Chinese Medicine, Tzu Chi University, Hualien 970, Taiwan

**Keywords:** bone mineral density, fibroblast growth factor 21, hemodialysis

## Abstract

Recent evidence has indicated that fibroblast growth factor 21 (FGF21) regulates longitudinal bone growth, with increased FGF21 levels leading to bone loss. The present study evaluated the relationship between bone mineral density (BMD) and serum FGF21 levels in patients undergoing hemodialysis (HD). We analyzed blood samples from 95 patients undergoing HD and measured BMD using dual-energy X-ray absorptiometry of the lumbar vertebrae (L2–L4). Serum FGF21 concentrations were determined using a commercially available enzyme-linked immunosorbent assay kit. Thirteen (11.6%) patients were found to have osteoporosis, 27 (28.4%) osteopenia, and 57 patients had normal BMD. Advanced age and decreased body mass index, height, body weight, waist circumference, and triglyceride level were associated with lower lumbar T-scores, as were increased alkaline phosphatase, urea reduction rate, fractional clearance index for urea, and FGF21 levels. Low log-FGF21, increased body mass index, increased pre-HD body weight, and increased logarithmically transformed triglycerides (log-TG) were found to be significantly and independently associated with lumbar BMD by multivariate forward stepwise linear regression analysis with adjustment for significant confounders. We conclude that high serum FGF21 level is negatively associated with BMD in patients undergoing HD.

## 1. Introduction

Chronic kidney disease–mineral bone disorder (CKD-MBD) is a common systemic disorder which occurs in the context of end-stage renal disease (ESRD) and manifests as mineral metabolism dysregulation, damage to bone structure, and vascular calcification [1]. As CKD progresses, the elevation of serum fibroblast growth factor 23 (FGF23) leads to suppression calcitriol production, consequently inducing hypocalcaemia and stimulating parathyroid hormone (PTH) secretion, ultimately resulting in decreased bone mineral density (BMD) [2]. This is associated with severe complications such as fracture, stroke, cardiovascular disease, and mortality [1,2]. Prior to beginning dialysis, 50% of patients with CKD will experience fracture [3,4]. Furthermore, patients with ESRD or young patients with CKD are at a higher risk of fractures [5].

Fibroblast growth factor 21 (FGF21) is a peptide hormone mainly secreted by the liver and brown adipose tissue [6], with potential clinical utility as a therapeutic approach for metabolic diseases due to its enhancement of insulin sensitivity and weight loss [7,8,9]. One study on mice showed that weekly injection of FGF21 increases bone resorption and decreases bone formation [10]. In rats fed a high-fat diet, daily injections of FGF21 reduced trabecular volumetric BMD, bone volume, and thickness of the tibia [11]. Another study indicated that systemic FGF21 downregulates bone formation during lactation in mice [12]. However, the relationship between FGF21 and human bone remains unclear. Given that FGF21 might influence BMD, our study aimed to identify the association between FGF21 with BMD in patients undergoing hemodialysis (HD).

## 2. Materials and Methods

### 2.1. Patients

We invited all patients in our HD unit above 50 years of age, undergoing 4 h dialysis three times a week for more than 3 months between June 2015 and August 2015 to participate in the study. Exclusion criteria were active infection, pulmonary edema, acute exacerbation of heart failure, acute myocardial infarction, malignancy, regular use of osteoporosis drugs (teriparatide, bisphosphonates, estrogen), and history of lumbar vertebrae surgery. The study and all its protocols were approved by the Protection of Human Subjects Institutional Review Board of Tzu-Chi University and Hospital (IRB106-62-B). The patients were excluded if they did not provide the written informed consent. The Kt/V and urea reduction ratio (URR) were measured using the single-compartment dialysis urea kinetic model.

### 2.2. Anthropometric Analysis

Body height was measured to the nearest 0.5 cm. Waist circumference was measured at the smallest point between the lower rib margin and the iliac crest, to the nearest 0.5 cm. Pre- and post-HD body weights were recorded to the nearest 0.5 kg with the patient in light clothing and without shoes. Body mass index (BMI) was calculated as weight (kg) divided by height squared (m). A single operator performed all the measurements [13,14,15].

### 2.3. Biochemical Analyses

Before HD, 5 mL of blood was collected from each patient and immediately centrifuged at 3000× g for 10 min. Serum samples were stored at 4 °C within 1 h of collection. We measured serum levels of total calcium, phosphorus, alkaline phosphatase (ALP), albumin, globulin, creatinine (Cre), blood urea nitrogen, glucose, total cholesterol (TCH), and triglycerides (TG) on an autoanalyzer (Siemens Advia 1800; Siemens Healthcare GmbH, Henkestr, Germany). Intact parathyroid hormone levels (iPTH) were tested using an autoanalyzer (Siemens Advia Centaur XP Immunoassay System; Siemens Healthcare GmbH). Serum intact FGF21 levels were measured using an enzyme-linked immunosorbent assay (ELISA) kit (Phoenix Pharmaceuticals, Inc. Burlingame, CA, USA). Briefly, the human FGF21 in the sample or in the standard solution can bind to the capture antibody immobilized in the wells. A standard curve of human FGF21 with known concentration can be established accordingly. The human FGF21 with unknown concentration in samples can be determined by extrapolation to this standard curve. Reactions were quantified by absorbance optical density using an automated ELISA reader (Sunrise, Tecan Co., Grödingen, Austria) at 450 nm wavelength. The intra-assay and inter-assay coefficient of variation in the measurement for FGF21 level was 3.5% and 5.2%, respectively.

### 2.4. Bone Mineral Density Measurement

Patients underwent BMD examination after blood sampling. Lumbar vertebrae (L1–L4) BMD was measured using dual-energy X-ray absorptiometry (QDR 4500; Hologic Inc., MA, USA). The T-score was defined as the number of standard deviations (SDs) from the mean BMD of gender-matched young control subjects, while Z-scores report the SDs matched by gender, age, weight, and ethnicity. According to World Health Organization criteria, osteoporosis was defined as a lumbar BMD T-score of ≤ −2.5 and osteopenia as T-score of −1.0 to > −2.5 [14].

### 2.5. Statistical Analysis

All statistical analyses were carried out using SPSS for Windows (version 19.0; SPSS Inc., Chicago, IL, USA). Data distribution was evaluated using the Kolmogorov–Smirnov test; normally distributed data are expressed as means ± SD, while non-normally distributed data are expressed as medians and interquartile ranges. Categorical data are expressed as the number of patients and was analyzed using the χ^2^ test. The significance of differences between groups (normal, osteopenia, and osteoporosis) was determined using the Kruskal–Wallis test for parameters with non-normal distributions and one-way analysis of variance for normally distributed data. The duration of HD and levels of TG, glucose, ALP, iPTH, and FGF21 exhibited skewed, non-normal data distributions. Therefore, these were transformed to the logarithm to the base 10. After transformation, log-HD duration, log-TG, log-glucose, log-ALP, log-iPTH, and log-FGF21 became normally distributed. Clinical variables that were correlated with lumbar BMD in patients undergoing HD were evaluated by univariable linear regression analysis and multivariate forward stepwise regression analysis. A two-tailed *p*-value of <0.05 was considered statistically significant.

## 3. Results

In total, 95 patients using the same high-flux polysulfone disposable artificial kidney (FX class dialyzer; Fresenius Medical Care, Bad Homburg, Germany) were enrolled (50 males and 45 females). The subjects ranged in age from 50 to 85 years. Table 1 shows baseline demographics and laboratory data classified by lumbar BMD group (normal, *n* = 57; osteopenia, *n* = 27; osteoporosis, *n* = 11). Advanced age (*p* = 0.001), low body height (*p* = 0.002), low pre- and post-HD body weight (*p* < 0.001), low BMI (*p* = 0.008), small waist circumference (*p* = 0.005), low serum TG level (*p* = 0.027), high serum ALP level (*p* = 0.022), high URR (*p* = 0.009), high Kt/V (*p* = 0.015), and high serum FGF21 level (*p* < 0.001) were found to be associated with lower lumbar T-score.

Table 2 details the lumbar BMD values according to sex, comorbidities of diabetes mellitus (DM) and hypertension, and use of anti-hypertensive agents. The BMD was significantly lower among females than males (*p* = 0.001). Lumbar BMD did not differ statistically according to presence of DM or hypertension or the use of ACEi, angiotensin receptor blockers, β-blockers, calcium-channel blockers, statins, or fibrates.

We further examined the correlations of lumbar BMD with several factors by univariable linear analysis (Table 3) and found height (*r* = 0.423, *p* < 0.001), pre-HD body weight (*r* = 0.423, *p* < 0.001), post-HD body weight (*r* = 0.420, *p* < 0.001), waist circumference (*r* = 0.223, *p* = 0.030), BMI (*r* = 0.269, *p* = 0.008), log-TG (*r* = 0.315, *p* = 0.002), and serum Cre level (*r* = 0.228, *p* = 0.026) to be positively correlated; age (*r* = −0.354, *p* < 0.001), log-ALP level (*r* = –0.223, *p* = 0.030), total calcium level (*r* = −0.221, *p* = 0.032), URR (*r* = −0.302, *p* = 0.003), Kt/V (*r* = −0.299, *p* = 0.003), and serum FGF21 level (*r* = −0.477, *p* < 0.001) were found to be negatively correlated with lumbar BMD. Lastly, multivariate forward stepwise linear regression analysis of the siginificant variables (gender, age, height, pre-HD body weight, waist circumference, BMI, log-TG, Cre, log-ALP, total calcium, log-FGF21, URR, and Kt/V) showed low log-FGF21, high pre-HD body weight, and high log-TG to be associated with lumbar BMD in HD patients.

## 4. Discussion

The results presented here show that the serum levels of FGF21 are significantly negatively correlated with BMD in patients undergoing hemodialysis, as are age, ALP, URR, and Kt/V. In contrast, pre- and post-dialysis height and weight, BMI, waist circumference, and TG level were found to be positively associated with BMD.

To date, there is limited information on the relationship between FGF21 and bone density. Through animal experiments, Wang et al. demonstrated that FGF21 upregulates the secretion of a pro-osteoclastogenic liver hormone, which binds to osteoclast precursors and promotes osteoclast differentiation. Similarly, to this receptor activator of nuclear factor-κB ligand (RANKL)-stimulated effect, FGF21 is known to induce bone resorption [16]. Furthermore, Wei et al. proposed that FGF21 potentiates the activity of peroxisome proliferator-activated receptor γ (PPAR-γ), consequently stimulating adipogenesis and inhibiting osteoblastogenesis of bone marrow mesenchymal stem cells [17]. Thus, FGF21 causes bone loss in rodents either by increasing osteoclasts or decreasing osteoblasts.

With regards to human studies, a cross-sectional study involving patients with type 2 DM revealed FGF21 to be negatively associated with procollagen type 1 amino-terminal propeptide (P1NP), a biomarker of bone formation [18]. Furthermore, a positive correlation has been reported between FGF21 and bone resorption biomarkers in patients with human immunodeficiency virus (HIV) [19]. While these findings are consistent with those of the present study, FGF21 has been reported to be positively associated with BMD in women, mostly premenopausal, from a small observational study [20]. The discrepancy may be attributed to differences in estrogen levels; estradiol regulates the physiological function of brown adipose tissue (BAT) [21,22], and FGF21 is primarily secreted from BAT. The mean age of participants in the present study was over fifty years old, and most of women were post-menopausal.

Alkaline phosphatase is a marker of bone turnover and vascular calcification in patients with CKD and was found to be negatively correlated with BMD in our study. A cross-sectional study involving 157 patients on HD in California reported the same result [23]. In a recent longitudinal study, this negative correlation was observed to extend to 24 months [24].

We identified a significant negative correlation between BMD and Kt/V, which are parameters of HD clearance. Another study found a similar correlation with near significance [23], and reported that patients with high BMI, which is associated with improved bone density, had higher volume distribution in the Kt/V calculation, thus a lower Kt/V level. This led to the bias of higher clearance ratio being associated with more severe bone loss. However, there is little information available regarding the effect of dialysis clearance on BMD, and the precise relationship between URR, Kt/V, and BMD remains to be clarified.

Overall, we identified a negative correlation between BMD and age, which reflects age-related osteoporosis, and BMD was significantly lower among postmenopausal females than males due to estrogen deficiency [25]. Parameters associated with higher body mass such as height, weight, BMI, and waist circumference were found to have a positive correlation with BMD. Increased lean body mass increases mechanical loading, which has positive impacts in bone formation [26]. However, increased BMI above normal may still have positive effect on BMD that may disappear in obesity [27]. Visceral obesity and systemic inflammation by higher BMI appear to impair the protection of weight on bone, leading to bone loss [28].

PTH plays a major role in the regulation of bone remodeling. Levels of iPTH have been reported to be negatively correlated with BMD among postmenopausal women [29,30]. In the context of ESRD, PTH represents the spectrum between high turnover status and adynamic bone disease. In the present study, iPTH was not found to correlate with BMD.

There are some limitations to our study which should be acknowledged. First, all patients were Taiwanese individuals who were undergoing hemodialysis, therefore the results may not be generalizable to other races or patients with CKD before renal replacement therapy. Second, serum FGF21 concentration gradually increases as estimate glomerular filtration rates (eGFRs) decline from early CKD to ESRD. Compared to the healthy population, the FGF21 levels were elevated 10 to 20 fold in ESRD patients. Beyond the renal clearance, accumulation of FGF21 may contribute by insulin resistance, chronic inflammation, and increased oxidative stress in uremic circumstances [31,32]. However, our study did not measure insulin resistance, inflammation, and oxidative stress. Furthermore, BMD was only measured in the lumbar region and no femoral measurements were taken. The BMD level may be overestimated due to aortic calcification. Moreover, since this was cross-sectional study, it was impossible to evaluate causal links between FGF21 and BMD.

To our best of our knowledge, this is the first study to investigate the relationship between FGF21 and BMD in hemodialysis populations. It provides a new approach for the prediction of comorbidities and prognosis of CKD-MBD using FGF21. However, confirming the negative association between FGF21 and BMD is only the first step, and future cohort studies or randomized control trials are needed to confirm the nature of the cause and effect relationship. Furthermore, multicenter studies are required in order to strengthen the generalizability of our results.

## 5. Conclusions

Our study demonstrates that serum FGF21 levels are significantly negatively associated with BMD, while BMI, pre-HD body weight, and serum TG levels are significantly positively associated with BMD in patients undergoing HD.

## Figures and Tables

**Table 1:** Baseline characteristics by categories of bone density in our study.

Characteristics	All Patients (*n* = 95)	Normal (*n* = 57)	Osteopenia (*n* = 27)	Osteoporosis (*n* = 11)	*p* Value
Age (years)	66.29 ± 9.98	63.65 ± 8.84	70.85 ± 8.55	68.82 ± 6.82	0.001 *
Hemodialysis duration (months)	50.28 (21.60–104.64)	47.52 (19.26–96.00)	55.68 (24.24–105.00)	89.04 (35.40–190.80)	0.093
Height (cm)	159.86 ± 8.24	162.23 ±7.32	156.67 ± 7.96	155.45 ± 9.62	0.002 *
Pre-HD body weight (kg)	63.54 ± 14.21	67.54 ± 13.77	60.22 ± 13.16	50.96 ± 9.54	<0.001 *
Post-HD body weight (kg)	61.49 ± 13.83	65.32 ± 13.26	58.43 ± 13.12	49.13 ± 9.43	<0.001 *
Waist circumference (cm)	91.64 ± 11.70	93.82 ± 11.76	91.15 ± 9.54	81.55 ± 11.65	0.005 *
Body mass index (kg/m^2^)	24.98 ± 4.74	25.88 ± 4.80	24.62 ± 4.57	21.18 ± 2.60	0.008 *
Bone mineral density (g/cm^2^)	0.90 ± 0.20	1.02 ± 0.14	0.75 ± 0.09	0.60 ± 0.08	<0.001 *
Lumbar T-score	−0.69 ± 1.49	0.27 ±1.02	−1.81 ± 0.51	−2.92 ± 0.39	<0.001 *
Lumbar Z-score	0.21 ± 1.25	0.84 ± 0.92	−0.37 ± 0.92	−1.57 ± 1.01	<0.001 *
Systolic blood pressure (mmHg)	142.57 ± 27.60	144.79 ± 26.34	144.37 ± 29.20	126.64 ± 27.25	0.125
Diastolic blood pressure (mmHg)	75.85 ± 14.95	77.30 ± 15.21	76.93 ± 14.97	65.73 ± 9.83	0.056
Total cholesterol (mg/dL)	141.56 ± 33.92	138.84 ± 36.04	145.33 ± 28.94	146.36 ± 35.47	0.636
Triglyceride (mg/dL)	112.00 (84.00–176.00)	127.00 (94.00–206.00)	105.00 (87.00–150.00)	83.00 (58.00–107.00)	0.027 *
Glucose (mg/dL)	138.00 (110.00–185.00)	143.00 (112.50–196.00)	132.00 (110.00–185.00)	121.00 (103.00–136.00)	0.059
Albumin (mg/dL)	4.11 ± 0.42	4.14 ± 0.32	4.12 ± 0.41	3.94 ± 0.79	0.355
Globulin (mg/dL)	3.15 ± 0.57	3.12 ± 0.48	3.18 ± 0.72	3.21 ± 0.61	0.820
Blood urea nitrogen (mg/dL)	59.23 ± 14.83	58.72 ± 13.67	59.56 ± 15.80	61.09 ± 19.11	0.883
Creatinine (mg/dL)	9.25 ± 1.98	9.62 ± 1.93	8.89 ± 1.96	8.27 ± 1.98	0.061
Alkaline phosphatase (U/L)	79.00 (63.00–107.00)	75.00 (61.00–97.50)	86.00 (72.00–124.00)	107.00 (79.00–127.00)	0.022 *
Total calcium (mg/dL)	9.00 ± 0.70	8.85 ± 0.65	9.22 ± 0.67	9.19 ± 0.93	0.054
Phosphorus (mg/dL)	4.57 ± 1.23	4.68 ± 1.13	4.53 ± 1.42	4.12 ± 1.25	0.385
Intact parathyroid hormone (pg/mL)	192.90 (58.50–354.10)	184.10 (68.10–272.80)	286.70 (56.70–466.70)	283.50 (58.50–341.10)	0.347
Fibroblast growth factor 21 (pg/mL)	998.24 (604.73–2239.39)	753.49 (493.13–1369.95)	1530.79 (873.18–2392.24)	3475.84 (3289.67–4099.43)	<0.001 *
Urea reduction rate	0.73 ± 0.04	0.72 ± 0.04	0.74 ± 0.04	0.76± 0.03	0.009 *
Kt/V (Gotch)	1.33 ± 0.16	1.29 ± 0.15	1.37 ± 0.16	1.42 ± 0.14	0.015 *

Values for continuous variables given as means ± standard deviation and test by one-way analysis of variance; variables not normally distributed given as medians and interquartile range and test by Kruskal–Wallis analysis. * *p* < 0.05 was considered statistically significant after Kruskal–Wallis analysis or one-way analysis of variance. HD, hemodialysis; Kt/V, fractional clearance index for urea.

**Table 2:** Clinical characteristics and lumbar bone mineral density levels of the 95 hemodialysis patients.

Characteristic		Number (%)	Lumbar BMD (g/cm^2^)	*p* Value
Gender	Male	50 (52.6)	0.96 ± 0.20	0.001*
	Female	45 (47.4)	0.83 ± 0.18	
Diabetes	No	47 (49.5)	0.86 ± 0.20	0.086
	Yes	48 (50.5)	0.93 ± 0.20	
Hypertension	No	49 (51.6)	0.90 ± 0.20	0.877
	Yes	46 (48.4)	0.89 ± 0.21	
ACE inhibitor or ARB use	No	73 (76.8)	0.90 ± 0.20	0.820
	Yes	22 (23.2)	0.91 ± 0.22	
β-blocker use	No	63 (66.3)	0.89 ± 0.22	0.662
	Yes	32 (33.7)	0.91 ± 0.17	
CCB use	No	64 (67.4)	0.89 ± 0.20	0.547
	Yes	31 (32.6)	0.92 ± 0.20	
Statin use	No	78 (82.1)	0.89 ± 0.20	0.702
	Yes	17 (17.9)	0.91 ± 0.22	
Fibrate use	No	84 (88.4)	0.90 ± 0.20	0.853
	Yes	11 (11.6)	0.89 ± 0.24	

Data are expressed as means ± standard deviation and test by Student’s *t*-test. * *p* < 0.05 was considered statistically significan. BMD, bone mineral density; ARB, angiotensin-receptor blocker; ACE, angiotensin-converting enzyme; CCB, calcium-channel blocker.

**Table 3:** Correlation of lumbar bone mineral density levels and clinical variables by simple regression or multivariable linear regression analyses among the 95 hemodialysis patients.

Variables	Lumbar Bone Mineral Density (g/cm^2^)				
	Univariate Regression		Multivariable Regression		
	*r*	*p* Value	Beta	Adjusted R^2^ Change	*p* Value
Age (years)	−0.354	<0.001 *	–	–	–
Log-HD duration (months)	−0.133	0.200	–	–	–
Height (cm)	0.423	<0.001 *	–	–	–
Pre-HD body weight (kg)	0.423	<0.001 *	0.616	0.117	0.001 *
Waist circumference (cm)	0.223	0.030 *	–	–	–
Body mass index (kg/m^2^)	0.269	0.008 *	0.396	0.028	0.028 *
Systolic blood pressure (mmHg)	0.078	0.453	–	–	–
Diastolic blood pressure (mmHg)	0.085	0.415	–	–	–
Total cholesterol (mg/dL)	0.080	0.442	–	–	–
Log-Triglyceride (mg/dL)	0.315	0.002 *	0.268	0.036	0.003 *
Log-Glucose (mg/dL)	0.159	0.123	–	–	–
Albumin (mg/dL)	0.169	0.101	–	–	–
Globulin (mg/dL)	−0.059	0.571	–	–	–
Blood urea nitrogen (mg/dL)	−0.032	0.756	–	–	–
Creatinine (mg/dL)	0.228	0.026 *	–	–	–
Log-Alkaline phosphatase (U/L)	−0.223	0.030 *	–	–	–
Total calcium (mg/dL)	−0.221	0.032 *	–	–	–
Phosphorus (mg/dL)	0.102	0.327	–	–	–
Log-iPTH (pg/mL)	−0.122	0.240	–	–	–
Log-FGF21 (pg/mL)	−0.477	<0.001 *	−0.392	0.219	<0.001 *
Urea reduction rate	−0.302	0.003 *	–	–	–
Kt/V (Gotch)	−0.299	0.003 *	–	–	–

Data of HD duration, triglyceride, glucose, alkaline phosphatase, iPTH, and FGF21 showed skewed distribution and therefore were log-transformed before analysis. Analysis of data was done using the univariate linear regression analyses or multivariate stepwise linear regression analysis (adapted factors were female, age, height, pre-HD body weight, waist circumference, BMI, log-TG, creatinine, log-alkaline phosphatase, total calcium, log-FGF21, urea reduction rate, and Kt/V). * *p* < 0.05 was considered statistically significant. HD, hemodialysis; iPTH, intact parathyroid hormone; FGF21, fibroblast growth factor 21; Kt/V, fractional clearance index for urea.
